# Efficacy and safety of the sofosbuvir/velpatasvir combination for the treatment of patients with early mild to moderate COVID-19

**DOI:** 10.1038/s41598-022-09741-5

**Published:** 2022-04-06

**Authors:** Vincenzo Messina, Riccardo Nevola, Antonio Izzi, Pellegrino De Lucia Sposito, Aldo Marrone, Roberto Rega, Raffaele Fusco, Paolina Lumino, Luca Rinaldi, Pasqualina Gaglione, Filomena Simeone, Ferdinando Carlo Sasso, Paolo Maggi, Luigi Elio Adinolfi

**Affiliations:** 1 Infectious Disease Unit, Azienda Ospedaliera Di Caserta S. Anna e S. Sebastiano, Caserta, Italy; 2 Department of Advanced Medical and Surgery Science, Internal Medicine Covid Center, University of Campania Luigi Vanvitelli, 80100 Naples, Italy; 3 Infectious Diseases, 3Rd Covid Center Ospedale Cotugno, Naples, Italy; 4 COVID Center, Ospedale Di Maddaloni, Caserta, Italy

**Keywords:** Virology, Target validation

## Abstract

SARS-CoV-2 is still a health problem worldwide despite the availability of vaccines. Therefore, there is a need for effective and safe antiviral. SARS-CoV-2 and HCV necessitate RNA-dependent RNA polymerase (RdRp) for replication; therefore, it has been hypothesized that RdRp inhibitors used to treat HCV may be effective treating SARS-CoV-2. Accordingly, we evaluated the effect of the sofosbuvir/velpatasvir (SOF/VEL) combination in early SARS-CoV-2 infection. A multicenter case–control study was conducted, enrolling 120 patients with mild or moderate COVID-19, of whom 30, HCV coinfected or not, received SOF/VEL tablets (400/100 mg) once daily for 9 days within a median of 6 days from the beginning of infection and 90 controls were treated with standard care. The primary endpoint was the effect on viral clearance, and the secondary endpoint was the improvement of clinical outcomes. Nasal swabs for SARS-CoV-2 by PCR were performed every 5–7 days. Between 5–14 days after starting SOF/VEL treatment, SAS-CoV-2 clearance was observed in 83% of patients, while spontaneous clearance in the control was 13% (p < 0.001). An earlier SARS-CoV-2 clearance was observed in the SOF/VEL group than in the control group (median 14 vs 22 days, respectively, p < 0.001) also when the first positivity was considered. None of the patients in the SOF/VEL group showed disease progression, while in the control group, 24% required more intensive treatment (high flow oxygen or noninvasive/invasive ventilation), and one patient died (p < 0.01). No significant side effects were observed in the SOF/VEL group. Early SOF/VEL treatment in mild/moderate COVID-19 seems to be safe and effective for faster elimination of SARS-CoV-2 and to prevent disease progression.

## Introduction

SARS-CoV-2, is a virus member of the Orthocoronavirinae subfamily and is responsible for the COVID-19 pandemic emergency. Remdesivir (RDV) is actually the only antiviral drug authorized for emergency use by the FDA for the treatment of COVID-19 based on data showing a possible faster recovery time, but it must be administered intravenously, does not work in everyone^1,2^ and recently WHO panel is recommending against it use because there is no evidence that the drug improves mortality, reduces initiation of ventilation, and duration of hospital stay^3^.

Although today we have effective vaccines, for various reasons, including the appearance of viral variants, the emergency/urgency state persists worldwide. Therefore, the need for effective antivirals remains a priority. The development of a new antiviral therapy generally takes many years^4^. One possible strategy is to verify whether the repurposing of already available antiviral compounds could be effective in inhibiting the replication of SARS-CoV-2. In this view, it is important to emphasize that coronaviruses and hepatitis C virus (HCV) are both positive-sense single-stranded RNA viruses^5,6^ requiring an RNA-dependent RNA polymerase (RdRp) for genome replication and transcription. Therefore, RdRp, which is highly conserved at the amino acid level in the active site, is one of the pivotal targets for effective antiviral treatment. In an in vitro model, sofosbuvir tightly binds SARS-CoV-2 RdRp, reducing the function of the protein and leading to viral eradication^6,7^. Thus, it has been proposed that nucleotide and nucleoside analogs that inhibit polymerases comprise a possible group of antiviral agents for COVID-19 treatment^7,8^.

The combination sofosbuvir/velpatasvir (SOF/VEL) is a potent inhibitor of RdRp and is safe and used worldwide for the treatment of HCV infection. SOF works by prevalently blocking NS5B and VEL by blocking NS5A. Experimental studies have shown that SOF binds the SARS-CoV-2 RdRp protein by blocking its function and underlining its potential therapeutic role^8,9^ and that VEL, in a virtual molecular model screening for readily available drugs, was considered an attractive molecule for therapeutic use against the major protease (Mpro) of SARS-CoV-2, therefore creating the molecular basis for sequential antiviral treatment along the SARS-CoV-2 life cycle^10^. On this basis, it has been hypothesized that the SOF/VEL combination has a role in the treatment of SARS-CoV-2 infection^8,9,11^. Therefore, we aimed to test the hypothesis that SOF/VEL treatment could be effective in rapidly clearing SARS-Covid-2 infection, thus avoiding disease progression.

In this study, we evaluated the safety and effects of SOF/VEP treatment on SARS-CoV-2 clearance times and clinical outcomes in patients with early mild or moderate COVID-19 coinfected or not with HCV.

## Patients and methods

### Patients

The patients were enrolled in 4 COVID Centers of Southern Italy, in the Campania Region: Caserta, Maddaloni and Naples. Patients with COVID-19 infection who were asymptomatic, paucisymptomatic or had mild pneumonia at presentation were enrolled in this study. A paucisymptomatic/mildly ill patient was defined as having symptoms of moderate dry cough, fever below 37.5 °C, general feeling of fatigue; muscle aches and possibly loss of taste and smell and moderate diarrhea, but having no wheezing or significant abnormal chest imaging; while for moderate disease, patients who exhibited lower respiratory tract disease but had ≥ 94% oxygen saturation (SpO_2_) in ambient air were considered. Severe disease included individuals who had an SpO_2_ < 94% in ambient air, a PaO_2_/FiO_2_ ratio < 300 mmHg, a respiratory rate > 30 breaths/min, or pulmonary infiltrates > 50% (https://www.covid19treatmentguidelines.nih.gov/o). Patient inclusion criteria were: men or women aged 18 years or older enrolled in the study with prior informed consent. For the female participant, pregnancy was ruled out before enrolment. Readiness to take study drugs or to comply with all study procedures, including repeated nasal/throat swab, and to be able to provide written and oral informed consent was assessed prior to enrollment.

The patient exclusion criteria were: serious respiratory failure with a respiratory frequency ≥ 25 breaths/min and/or a PaO_2_/FIO_2_ (P/F) ratio < 300. Critical patients with one of the following conditions were excluded: shock; need of intensive care; severe liver dysfunction (Cirrhosis Child-Turcotte Pugh C); need of dialysis treatment or GFR ≤ 30 mL/min/1.73 m^2^; or serious neurological and mental disorders. In addition, patients taking drugs that could interfere with the investigational drugs, individuals with known hypersensitivity to the study drugs and people already treated with other investigational antiviral drugs were excluded.

### Study design

A case–control (matched:1–3) study was conducted. The outcomes of each enrolled SOF/VEL-treated plus standard of care (SOC) case were compared with those of 3 consecutively enrolled controls treated with SOC that were matched for age, sex, duration of infection and comparable clinical condition. All patients (treated and control) received similar SOCs. SOC consisted of symptomatic therapy with antipyretics or FANS as needed. For bedridden patients a low molecular weight heparin prophylaxis was given. If patients showed desaturation during hospitalization, O_2_ supplementation and dexamethasone treatment was started. If patients had bacterial superinfection, antibiotic therapy was initiated. Active comorbidities were treated according to their respective guidelines.

### Methods

The presence of SARS-CoV-2 was assessed by real-time RT-PCR, which identifies the RdRp and E genes of SARS-CoV-2 by a *COBAS* 6008 Roche.

### Treatment

The treated group received SOF/VEL tablets (400/100 mg) once a day for 9 days plus SOC, whereas the control group received SOC only. In non-HCV positive COVID-19 patients, treatment with SOV/VEL was discontinued after 9 days of treatment, while treatment of patients co-infected with HCV continued for 12 weeks. In non-HCV-coinfected patients, the drug was authorized by the local competent committee for off-label use, and all patients gave signed informed consent.

### Treatment outcomes

The endpoints were both virologic and clinical outcomes. The primary outcome was virologic, i.e., the time of SARS-CoV-2 clearance. Such an endpoint was assessed by nasal/throat swabs with RT-PCR performed at baseline day "0" and repeated before the start of treatment (pretreatment) and at posttreatment days 5,10,15,20,25. In the control group, the nasal/throat swab was repeated every 5 days. However, if the results were dubious, PCR was repeated between the intervals. The secondary outcomes were clinical, in particular progression of disease such as need for high flow nasal cannula (HFNC) oxygenation or mechanical ventilation, mortality at 28 days and duration of hospital stay. The main clinical and laboratory parameters concerning the functionality of the major organs were monitored and recorded.

### Statistics

Results are expressed as median and range. The Mann–Whitney U test was used to evaluate differences between groups. The chi-square test with Yates correction was used to evaluate differences between categorical variables of the two groups. The time of SARS-CoV-2 clearance between groups was evaluated by Kaplan–Meier curves and the log rank test for individual differences between groups. A p value of 0.05 was assumed to denote significance.

### Ethical statement

All methods were carried out in accordance with relevant guidelines and regulations. The study was approved by the local ethics committee “Campania Nord” (Study: An. Vi. Cov19. Registered on 17 April 2021 on the Italian platform of the Ethics Committee: number CECN:1553)

## Results

The diagnosis of SARS-CoV-2 infection was made on at least 2 different samples obtained by oral and nasal swabs using RT-PCR, of which one was on the day of initiation of SOF/VEL treatment. A total of 120 patients with SARS-CoV-2 infection were enrolled in the study: 30 patients were treated with SOF/VEL and SOC, and 90 as controls were treated with SOC. Among the treated cases, 60% were coinfected with SARS-CoV-2/HCV. Thirty percent of the patients treated with SOF/VEL and 31% of the controls were followed up as outpatients.

The main demographic and clinical characteristics of the SOF/VEL patients and SOC group are shown in Table 1. The demographic and clinical characteristics at presentation of the two groups were comparable. Among the SOF/VEL-treated group, 63% were male, the median age was 54 years (range 23–77), and the patients, at the time of starting treatment had a mild to moderate pneumonia or were asymptomatic or paucisymptomatic. Similarly, control patients treated with SOC had an initial mild to moderate clinical form of COVID-19 and were hospitalized in the same period of treated cases, consisting of 90 consecutive patients who had SARS-CoV-2 infection and were evaluated to establish the timing of spontaneous viral clearance and clinical outcome. The control group had an average age of 53 years, and 62% were male. Pre-existing associated diseases were present in the SOF/VEL group and control group in 58% and 68.8%, respectively. In the SOF/VEL group, 4/18 (22%) HCV-positive patients had cirrhosis, and 2 showed decompensated cirrhosis. The majority of patients had chest X-ray or computed tomography showing bilateral ground glass opacities (GGOs). The median duration of infection at hospitalization was similar for both groups.

**Table 1:** Demographic and clinical characteristics at presentation of the enrolled patients.

Patient's characteristics	SARS-CoV-2 SOF/VEL Treated	SARS-CoV-2 untreated Patients	p =
Number	30	90	
Male (%)	66	62	ns
Coinfected SARS-CoV-2/HCV, n	18	0	
Median Age, yrs (range)	54 (23–77)	53 (38–86)	ns
Fever and/or cough and/or anosmia, %	67	70	ns
**Radiology**			
Ground glassy opacity, %	70	77	ns
Pneumonia, %	6,6	8.8	ns
PaO_2_/FIO_2_ ratio, median range]	330 [280–438]	338 [282–448]	ns
Required O_2_ therapy, %	20	22	ns

ns = not significant.

**Figure 1:**
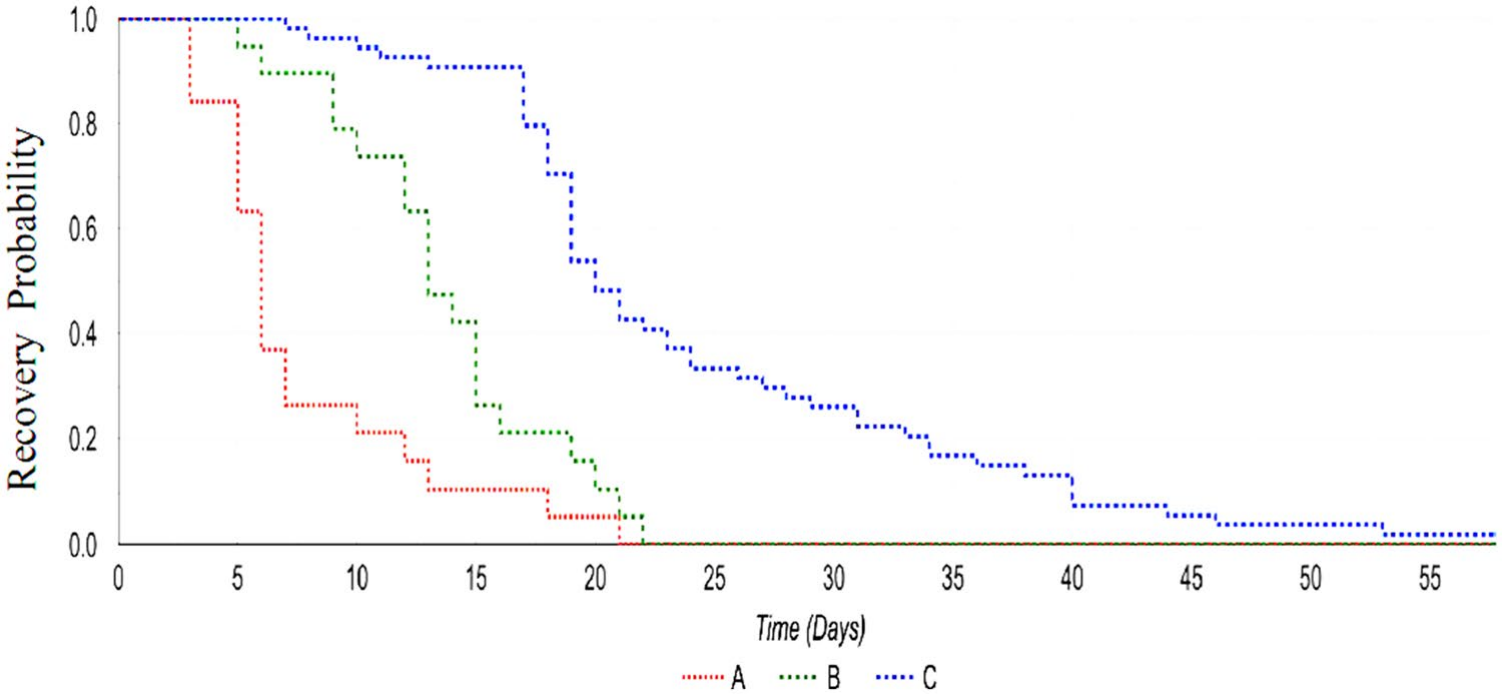
Kaplan–Meier curves of SARS CoV-2 infection clearance by SOF/VEL treatment (lines A,B) and spontaneously (line C; untreated control patients). Line (A): recovery time from infection starting from the first day of treatment with SOF/VEL. Lines (B,C) recovery time from infection starting from the first positive nasopharyngeal swab. Log-rang test: χ^2^ = 307.02, p < 0.0001.

Table 2 shows the time of SARS-CoV-2 clearance after beginning of SOV/VEL treatment of the 30 patients treated and of the control group. The median duration from diagnosis of infection and inclusion in the study was similar in the two groups (Table 2). The data shown in Table 2 and Fig. 1 demonstrate that in the SOF/VEL group, SARS-CoV-2 clearance was significantly faster than in the control group (p < 0.001). In particular, more than half (53%) of the SOF/VEL-treated patients cleared SARS-CoV-2 during the first 7 days of treatment, whereas it occurred in only 1.1% of control patients. Furthermore, 83% of SOF/VEL-treated patients eliminated the virus within 14 days of treatment, with respect to only 13% of SOC patients (Table 2). Figure 1 shows the Kaplan–Meier curves of SARS CoV-2 infection clearance in the different groups evaluated. The curves show that SARS CoV-2 infection clearance was significantly faster in the SOF/VEL treated group than SOC group (Log-rang test: χ^2^ = 307.02, p < 0.0001). An analysis of SARS-CoV-2 clearance time by SO/VEL between patients with HCV and without HCV showed no significant difference (data not shown).

We also observed important differences between the two groups in terms of both disease progression and outcome. In fact, none of the 30 patients treated required high-flow oxygen, or noninvasive or invasive ventilation, and, more importantly, no patient died. On the other hand, in the control group, 24% of patients had disease progression and required more intensive treatments (high flow oxygen or noninvasive/invasive ventilation), and one patient died (p < 0.01).

**Table 2:** SARS-CoV-2 clearance in patients treated with SOF/VEL and in untreated patients.

	SARS-CoV-2 SOF/VEL treated	SARS-CoV-2 Untreated Patients	P =
Number	30	90	
Duration of infection before antiviral treatment (days of PCR positive), median (range)	6 (3–13)	7 (4–14)*	ns
Days of SARS-CoV-2 Clearance by first positivity, median (range)	14 (6–23)	22 (7–55)	0.001
Days of SARS-CoV-2 Clearance by starting SOF/VEL, median (range)	6 (5–21)	22 (7–55)	0.0001
**SARS-CoV-2 clearance**			
≤ 7 days, %	50	1.1	0.001
≤ 14 days, %	83	13	0.001
≤ 21 days, %	93	38	0.001
≤ 27 days, %	100	67.3	0.001
**Clinical outcome**			
Disease progression, %	0	24	0.001
Mortality, %	0	1.1	ns

*Days of SARS-CoV-2 positivity before enrolled in the study.

*ns* not significant.

SOF/VEL treatment was safe, and no significant adverse events were observed.

## Discussion

Our data, although preliminary and obtained in a limited number of patients with mild to moderate COVID-19, suggest that the SOF/VEL antiviral combination, currently used for the treatment of hepatitis C, is safe and effective for the treatment of these forms of SARS-CoV-2 infection.

The data indicate that early administration of SOF/VEL can clear SARS-CoV-2 infection in a significant proportion of patients in a shorter time than untreated (Fig. 1), preventing the disease from worsening. Indeed, SOF/VEL was effective in inducing SARS-CoV-2 clearance within 7 days in half of the cases and in over 80% within 14 days; in contrast, in patients receiving SOC alone, spontaneous viral clearance was observed in 1.1% and 13% at 7 and 14 days, respectively (p < 0.001; Table 2).

To reinforce the data of our study are the positive results presented at recent Liver International Meeting^12^ by an Egyptian group that used another anti-HCV drug combination, sofosbuvir/ledispavir (SOF/LED), the latter drug very similar to velpatasvir, in 65 patients with mild/moderate COVID-2 disease compared with 62 control patients treated with SOC alone. The clearance of SARS-CoV-2 was assessed on days 5, 8, 11 and 14 post-beginning treatment with SOF/LED, which was found to be 36.9%, 66.2%, 80% and 83.1%, respectively, while in the control group, viral clearance resulted in 0%, 1.6%, 17.7% and 19.4% (p < 0.01), respectively. These data are perfectly superimposable on the data obtained in our study using SOF/VEL.

Although, the majority of patients with mild to moderate disease spontaneously recover, some may have disease progression; thus, studies demonstrating the effectiveness of an oral antiviral are important because such a drug can be used on an outpatient basis to prevent the spread of the infection and avoid the progression of disease. In this context, it should be emphasized that RDV, the only approved antiviral at the time of this study, must be administered intravenously and is reserved only for hospitalized patients who need oxygen therapy; therefore, it is not effective in reducing the early spread and progression of the infection in the general population. At present, new oral antivirals such as molnupiravir and paxlovid have been approved.

Further support for the use of nucleosides comes from a recent experimental molecular basis study demonstrating that sofosbuvir shows more protection against SARS-CoV-2 exonuclease activity than RDV, and the author concluded that the use of sofosbuvir in combination with other drugs, e.g., velpatasvir, in clinical trials for COVID-19 should be a more appropriate treatment^13^.

It could be of interest to report recent data from an experimental study using a human lung epithelial cell line infected with a clinical isolate of SARS-CoV-2 showing that the combination of SOF/VEL increased RDV potency of 25-fold in the elimination of SARS-CoV-2 from infected cells^14^. These experimental data coupled with the results of this study and other similar studies^12^ suggest the possible use of an association between SOF/VEL or SOF/LED and RDV to be evaluated in future clinical trials to improve antiviral treatment of SARS-CoV-2 infections.

It should be noted that our study has some limitations. In particular, due to the restrictions in Italy in the use of SOF/VEL to HCV patients only, the number of patients enrolled in the study was low which does not allow definitive conclusions to be drawn on the real efficacy of the antiviral, and further powered controlled studies are needed to better define the impact that this antiviral combination could have on COVID-19. However, this study, unlike all other studies except one^12^, has evaluated a solid end-point that is the effect on the elimination of virus and not only surrogate clinical end-points such as the time to discharge from the hospital which may be that can be conditioned by various factors such as the duration of prehospitalization illness and the pressure of the pandemic on hospital admissions. Furthermore, antiviral treatment in our study has been used in COVID-19 patients with early mild/moderate clinical conditions; therefore, it is not possible to know whether the same efficacy is maintained even in patients with advanced/severe COVID-19 disease, in which inflammatory and immunological phenomena prevail over direct viral action; therefore, in severe patients, it is necessary to carry out suitable controlled clinical studies. In this respect, an Iranian study^15^ conducted in a severe patient setting has just been published. Forty mild/severe COVID-19 patients treated with SOF/VEL and 40 with SOC were enrolled. The endpoints were clinical, and the results showed that SOF/VEL did not improve clinical status and did not reduce mortality in patients with severe COVID-19. The authors concluded that larger randomized clinical trials including more parameters are needed for an accurate assessment of the efficacy of SOF/VEL in patients with severe disease considering that the study included a very heterogeneous population in terms of pretreatment duration of infection, a low number of both patients and observed events and in particular the clearance of SARS-CoV-2 has not been evaluated. In contrast to the above Iranian study^15^, a recent meta-analysis^16^ of 3 studies that used the combination sofosbuvir/daclastavir (SOF/DCV), the latter of which was very similar to velpatasvir, in patients with moderate/severe COVID-19 concluded that antiviral treatment improved the survival and clinical recovery of these patients. In fact, these studies showed that patients treated with SOF/DCV had an improvement in clinical recovery within 14 days [risk ratio = 1.34 (95% CI 1.05–1.71), p = 0.020], an improved time to clinical recovery [risk ratio = 2.04 (95% CI 1.25–3.32), p = 0.004] and a lower all-cause mortality [risk ratio = 0.31 (95% CI 0.12–0.78), p = 0.013]. However, the combined sample size of these studies was relatively small since they included 176 patients; thus, it is not possible to draw definitive conclusions.

A large randomized multicenter study from Iran, available as a preprint on the Lancet^17^, with moderate/severe COVID-19 treated with SOF/DCV did not show any benefit on the primary endpoint of hospital discharge after 10 days; thus, the results are not consistent with previous smaller trials and meta-analyses^16^. However, the weaknesses of the study are the lack of virologic investigation after treatment, the short observation period established as end point and due to lack of hospital beds, patients who were deemed well enough were discharged quickly and some patients self-discharged once they felt better. Therefore, the authors concluded that considering the advanced stage of the disease of the included patients, SOF/DCV should be investigated in earlier stages of disease when the virus plays a fundamental role in the development and progression of the infection as opposed to when it happens in the advanced stage of the disease where the immunological, inflammatory and complication phenomena have a preponderant role on the outcome of the disease, and therefore the elimination of the virus at this stage may not change the outcome of the disease and the use of an antiviral is not an indication of primary importance since its effect may be irrelevant and inconstant.

In conclusion, the results of our study conducted with SOF/VEL, although preliminary and require further confirmation in a larger randomized trial, in agreement with the recently reported data with SOF/LED^12^, indicate that early treatment of patients with mild to moderate COVID-19 may be safe and effective for faster elimination of SARS-CoV-2 and in the prevention of progression and outcomes of COVID-19. The data also support the conclusions of other studies using SOF/VEL and SOF/DCV^15,17^ in the late stage of COVID-19 disease that invoke studies in the early stages of the disease when the damage is mainly related to the action of the virus, before the onset of the inflammatory/immunological cascade that characterizes severe forms of the disease. Furthermore, it must be emphasized that SOF/VEL as oral drug can be used in early infection in the outpatient setting, which may be important in helping to reduce the spread of infection, and hospitalization and avoid disease progression.
